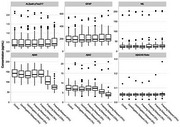# Effects of Processing Deviations on Plasma Concentrations of AD Biomarkers

**DOI:** 10.1002/alz70861_108816

**Published:** 2025-12-23

**Authors:** Kate Lange, Victoria J. Williams, Ralph Trane, Rachael E. Wilson, Ramiro Eduardo Rea Reyes, Beckie Jeffers, Elysse Keske, Cindy Jensen, Aaron Fredricks, Brittani J. Strait, Anne M Fischer, Nicole Cooke, Emma Henning, Pamela Herd, Michal Engelman, Sterling C Johnson, Henrik Zetterberg, Sanjay Asthana

**Affiliations:** ^1^ University of Wisconsin‐Madison, Madison, WI USA; ^2^ Wisconsin Alzheimer's Disease Research Center, University of Wisconsin‐Madison, School of Medicine and Public Health, Madison, WI USA; ^3^ University of Michigan, Ann Arbor, MI USA; ^4^ Wisconsin Alzheimer’s Disease Research Center, School of Medicine and Public Health, University of Wisconsin‐Madison, Madison, WI USA; ^5^ Wisconsin Alzheimer's Disease Research Center, School of Medicine and Public Health, University of Wisconsin‐Madison, Madison, WI USA

## Abstract

**Background:**

Blood‐based biomarkers have emerged as a promising tool for detecting the presence of Alzheimer’s disease (AD) pathology in vivo, offering a practical alternative to more costly and invasive biomarker approaches. However, as their use expands to research studies employing field‐based blood collection, it remains unclear how deviations from gold‐standard processing protocols that may arise in such resource limited collection environments stand to impact assay performance and validity. To address this concern, we systematically examined how delayed centrifugation and delayed freezing of blood samples impacts AD‐related biomarker concentrations as compared to gold‐standard processed samples that were frozen as close to draw time as possible.

**Method:**

Using plasma samples from 35 participants from the Wisconsin Registry for Alzheimer’s Prevention (WRAP) and the Wisconsin Longitudinal Study (WLS), aliquots underwent a set of within‐subject treatments: a reference aliquot that was processed according to gold‐standard protocols and frozen immediately at ‐80°C; delayed centrifugation at 1 hour and 2 hours beyond protocol; and delayed freezing where aliquots sat at room temperature for 2, 3, 23, and 47 hours beyond protocol. Plasma concentrations of five AD‐related biomarkers were subsequently assayed using the Quanterix SIMOA platform: ALZpath pTau217, GFAP, NfL, Aβ40, and Aβ42. For each biomarker, we calculated the within‐subject difference in average concentration levels between the non‐standard treatments and the sample processed according to gold‐standard protocols.

**Result:**

As shown in Figure 1, ALZpath pTau217, GFAP, and NfL all showed stable concentrations despite delayed centrifugation and delayed freezing, whereas Aβ40 and Aβ42 concentrations were particularly sensitive to delayed freezing. However, the rate of deterioration of Aβ40 and Aβ42 levels were similar such that the ratio of Aβ42/Aβ40 remains largely unaffected.

**Conclusion:**

Our results highlight the stability of plasma pTau217, NfL, and GFAP concentrations across sample processing protocol deviations, suggesting that these biomarkers are particularly well‐suited for use in low‐resource or field‐based settings. In contrast, we observed a notable decay in Aβ40 and Aβ42 concentration levels as a function of delayed freezing time, indicating concern for data validity in less controlled or reliable settings.